# Networks of Adversity in Childhood and Adolescence and Their Relationship to Adult Mental Health

**DOI:** 10.1007/s10802-022-00976-4

**Published:** 2022-11-04

**Authors:** Ayla Pollmann, Jessica Fritz, Edward Barker, Delia Fuhrmann

**Affiliations:** 1 Institute of Psychiatry, Psychology & Neuroscience, Department of Psychology, King’s College London, Addison House, Guy’s Campus, SE1 1UL London, UK; 2 Department of Psychiatry, University of Cambridge, Cambridge, UK; 3 Department of Clinical Psychology, Philipps-University Marburg, Marburg, Germany; 4 Institute of Psychiatry, Psychology and Neuroscience, Department of Psychology, King’s College London, Henry Wellcome Building for Psychology, Denmark Hill Campus, SE5 8AF, London, UK

**Keywords:** Development, Network analysis, Longitudinal cohort studies, Abuse, Trauma, ALSPAC

## Abstract

Adverse experiences before the age of eighteen are common and include diverse events ranging from sexual abuse to parental divorce. These stressful experiences have been linked to physical and mental health issues. Previous research has focused mainly on childhood adversity, such as experiences in the family environment. Little consideration has been given to adversities that may be particularly harmful in adolescence. To understand adolescents’ adverse experiences, this project used data from the Avon Longitudinal Study of Parents and Children (ALSPAC, total N = 14,901, N ≈ 1,200 − 10,000 per measure). We modelled interrelations of adversities in childhood (1–11 years) and adolescence (11–23 years) and examined adversity clusters using network analysis. We found two similar clusters in the childhood and adolescence networks: (1) direct abuse and (2) adverse family factors. We identified a third cluster of (3) educational and social adversities for adolescence. For both age groups, emotional abuse in the family environment was closely linked to mental health in early adulthood and most adversities were linked with depression in early adulthood. In adolescence, housing and academic issues and abuse by a romantic partner were particularly central to the network of adversities. Thus, we found commonalities and differences in the relevance of adverse experiences at different developmental stages. These findings highlight the need to develop age-dependent frameworks for adversity research and policymaking.

**Supplementary Information:**

The online version contains supplementary material available at 10.1007/s10802-022-00976-4.

## Networks of Adversity in Childhood and Adolescence and Their Relationship to Adult Mental Health

Approximately two-thirds of the population experiences at least one adversity before the age of eighteen (Merrick et al., 2018). These adversities include diverse experiences ranging from parental divorce to physical abuse (Petruccelli et al., 2019; Walsh et al., 2019). The field recognises a canonical set of “Adverse Childhood Experiences” (ACEs) based on Felitti et al., (1998), who categorised ACEs into “child abuse”, with psychological, physical, and sexual abuse, and “household dysfunction”, including parental and caregiver substance abuse or mental illness. There is compelling evidence that ACEs are linked to adverse outcomes during adulthood (Felitti et al., 2019; Muniz et al., 2019; Petruccelli et al., 2019; Slavich et al., 2019; Zhang et al., 2019). This includes psychopathologies like depression, eating disorders, substance abuse, and anxiety (Hovens et al., 2010; Kessler et al., 2010; Speranza et al., 2003; Witt et al., 2019), as well as well-being factors such as decreased life satisfaction (Hughes et al., 2016). The ACE framework has had a significant societal impact on childhood family and public health policy (Edwards et al., 2019): Early prevention and intervention have been critical in improving later life outcomes for children affected by adversities (Purewal Boparai et al., 2018; Srivastav et al., 2020).

Existing work on ACEs has been focused on early experiences like parental and caregiver adversity and family environments (Björkenstam et al., 2018; Petruccelli et al., 2019; Turner et al., 2020). This focus has inadvertently left another potential sensitive period for adversity neglected: Adolescence. As children grow into adolescents, they experience a range of social and biological changes that may lead to a second period of increased susceptibility to environmental influences after infancy and toddlerhood (Andersen & Teicher, 2008; Fuhrmann et al., 2015; Kolb, 2009).

Adolescence is not only a time of physical changes but also of social and environmental changes, including an increase in autonomy. As adolescents become more independent, the time they spend with family decreases and relationships outside the family environment tend to become increasingly influential.(De Goede et al., 2009) While adverse experiences in childhood often tend to occur in the family and caregiver context (Felitti et al., 1998), adolescents’ experiences are increasingly shaped by neighbourhoods, educational settings, and peer- and romantic relationships (Kiff et al., 2012; Mann et al., 2014; Slavich et al., 2019). While many and perhaps even most adversities can be experienced in both childhood and adolescence, initial evidence suggests that adversities such as bullying and peer social exclusion are especially detrimental during adolescence (Arseneault, 2017; Fuhrmann et al., 2019).

It has been suggested that there is an increasingly complex pattern of adverse experiences in adolescence, compared to early childhood, as co-occurrences of adversity exposures become more multifaceted with age (i.e., more variance in the types and patterns of experienced ACEs) (Grasso et al., 2016). However, traditional analysis approaches have been unable to capture these complexities; most ACE studies have calculated a cumulative risk score based on the number of adversities experienced and then investigated how these cumulative scores relate to later life outcomes (Danese & Lewis, 2022). These risk scores do not reflect the type, timing, or duration of the experienced adversities. The underlying assumption is that all adversities have a similar impact and interchangeable mechanisms (McLaughlin & Sheridan, 2016). This approach does not consider how adversities co-occur (Sheridan et al., 2020) and has limited capacity to capture the complexities and inter-relationships of life stressors in childhood and adolescence (Breuer et al., 2020; Slavich et al., 2019).

However, a complex system approach can be used to capture complexities in social science research. A complex system refers to a system greater than the sum of its parts (Meadows, 2009). A complex system can be dynamic as different components interact with each other. Using this approach in psychopathology, instead of analysing individual parts of a mental health disorder (e.g., a specific symptom), we can look at different aspects of a disorder interacting and changing over time (Hayes & Andrews, 2020). Network analysis as a complex system approach is designed to address issues of complexity in psychological research (Epskamp et al., 2018). Psychological research is inherently complex, as there are numerous potential sources and interactive relations in social and behavioural phenomena (Sanbonmatsu et al., 2021). One such phenomenon is adversity, as adverse experiences are multifaceted and highly interrelated (Dong et al., 2004).

Previous research has illustrated that adversities tend to co-occur (Bussemakers et al., 2019; Dong et al., 2004; Lacey et al., 2020). In other words, children who experience one kind of adversity are more likely to experience other types of adversities. Examining the clustering of ACEs can facilitate the identification of groups vulnerable to experiencing multiple adversities (Bussemakers et al., 2019). Moreover, particular combinations of adverse experiences might be related to specific adulthood outcomes (Anyigbo et al., 2021; Bussemakers et al., 2019). It is, therefore, crucial to not only understand the influence of individual adversities on later life outcomes; but to also understand (a) which and how adversities interact as well as (b) what types of adversities are particularly influential for the mental health in later life, when a multitude of adversities are considered at the same time (Lacey et al., 2020).

Network analysis is an excellent tool to visualise (partial-) correlations. It can be used to illustrate general patterns (such as clusters) of adversities, more nuanced relationships between particular adversities, as well as relationships between the adversities and mental health outcomes. Hodgdon et al., (2019) conducted one of the few studies that have examined the co-occurrence of different traumatic experiences and childhood adversities using network analysis. In this study, participants’ ages ranged from 4 to 18 years. The researchers determined different clusters of childhood trauma and childhood adversities, indicating that neglect and psychological maltreatment were most strongly associated with other trauma or abuse types. Another network study by Breuer et al., (2020) included an adult sample (with a retrospective adversity report). Here, the authors identified two ACEs clusters. The two clusters comprised direct (child maltreatment) and indirect traumatisation (household dysfunction). Both clusters were centred around the family context, highlighting the relevance of the family environment for early childhood. We will extend this work by (a) using a partially prospective, large sample, (b) a broader set of adversities (ACEs/AAEs) and (c) investigating two developmental periods: namely, childhood and adolescence.

We here apply network analysis to determine influential adversities in childhood and adolescence. We will evaluate the individual relationships between adversities as well as their relationships with mental health outcomes in early adulthood to assess the effect of each. We will assess the importance of each adversity in the network using centrality indices such as strength and expected influence coefficients (Hevey, 2018). While it is not possible to directly compare the networks in both age groups, as measures differed between the two developmental stages, we will explore which adversities are particularly closely related to each other in childhood and adolescence to establish whether there are distinct clusters of adversities in childhood and adolescence. This knowledge will help identify potential targets for translational research by highlighting which adversities are especially central during childhood, which during adolescence, and which during both developmental stages.

Previous network analyses of ACEs have mainly used retrospective measures. We will here leverage the strengths of a prospective longitudinal data set: the Avon Longitudinal Study of Parents and Children (ALSPAC, currently N = 14,901, with N ≈ 1,200 − 10,000 per measure, followed from the prenatal period until 27 years of age (Boyd et al., 2013; Fraser et al., 2013; Northstone et al., 2019). We will use the term classic adversities to refer to the ACE measures included in the ALSPAC, based on the landmark study by Felitti et al., (1998). Additionally, we included other adverse experiences relevant to adolescence based on Slavich et al., (2019). This project will contribute to a standardised framework for understanding *adverse adolescent experiences* (AAEs). Advancing our understanding of the topology of adversity during childhood and adolescence will inform translational research on prevention and intervention methods tailored to different developmental stages. In the long run, such work will contribute to establishing actionable insights for mental health policy and practice.

We hypothesised that (1) there will be distinct adversity clusters in childhood and adolescence. Also, we hypothesised that (2) there will be distinct influential adversities in childhood and adolescence. We expected that (3) there would be a relationship between classic adversities in childhood and adolescence and early adulthood mental health. Lastly, (4) we predicted that non-ACE adversities in adolescence (e.g., being bullied) would be related to mental health in adulthood.

## Methods

### Cohort

ALSPAC (Boyd et al., 2013; Fraser et al., 2013; Northstone et al., 2019) is a birth cohort study centred in Bristol, England, set up to investigate child development and health. Between 1991 and 1992, pregnant women were enrolled in the study. Their children were assessed throughout infancy, childhood, adolescence, and early adulthood. The study includes environmental, socioeconomic, lifestyle, genetic, and biological data. The sample used here consisted of 14,901 participants alive at year 1, including additional participants recruited at age 7. The data used in this study includes participants aged 1 to 23 years, with sample sizes per measure ranging from 1,200 to 10,000. Most of the sample had a white ethnic background (77.2%), a middle-class socioeconomic status (33.1%) and were male (51.2%). More detailed demographic information and prevalence rates of adversities and mental health issues can be found in Supplementary Tables 1 and 2. Ethical approval for the study was obtained from the ALSPAC Ethics and Law Committee and the Local Research Ethics Committees. Informed consent for the use of data collected via questionnaires and clinics was obtained from participants following the recommendations of the ALSPAC Ethics and Law Committee at the time.

## Measures

We investigated adverse experiences in childhood and adolescence. Age 1 to 11 was defined as childhood, while age 11 to 23 was defined as adolescence, based on the measures provided by the ALSPAC. Classic adversities were based on the seminal ACE study (for details, see (Felitti et al., 1998)). This includes exposure to psychological, mental, physical, and sexual abuse, parental mental illness, parental partner abuse, and parents’ criminality. Additional adverse adolescent experiences (AAEs) were based on the Stress and Adversity Inventory for Adolescence (STRAIN, Slavich et al., 2019). We included 11 out of 12 primary life domains used in the inventory (e.g., parent/guardian, legal/crime, treatment/health, education, reproduction, other relationships, death, housing, marital/partner, life-threatening situations, and work). The only life domain not included was financial difficulties, as we did not find a corresponding item in the available ALSPAC dataset. Response options of the variables can be found in Supplementary Table 2.

### Classic ACEs

Physical and emotional abuse and sexual abuse at age 0–11 and age 11–17 were assessed in the “Life at 22+” questionnaire at age 22 via self-report. Parental substance abuse, parental partner cruelty, parental criminality, and parental psychopathology was assessed in multiple questionnaires, predominantly via caregiver report:

During their child’s childhood, maternal substance abuse was examined at age 1 year and 9 months in the “Caring for a toddler” questionnaire completed by the mother. Carer substance abuse at the child’s age of 2 years and 9 months was assessed in the “Partner’s health, events and feelings” questionnaire, which the partner completed. During adolescence (at age 18 and 6 months), maternal substance abuse was examined with the “You and your Life” questionnaire completed by the mother. During childhood (at age 2 years and 9 months), partner cruelty was measured with the mother-completed questionnaire “Your health events and feelings”. During adolescence (the study child’s age was 11 years and 1 month), partner cruelty was examined with the “Lifestyle and health of mother” questionnaire, completed by the mother. When children were 1 year and 9 months old, mothers’ partner’s trouble with the law was assessed in the “A toddler in the house” questionnaire completed by the partner. During adolescence (at the age of 16), mothers’ partner’s crime was examined in the “Life of a 16 + Teenager” scale, as reported by the teenagers. Parental Psychopathology was assessed for both childhood and adolescence at the study child’s age of 18 years and 6 months. Mothers responded in the “You and your life” questionnaire.

### Adolescent Adverse Experiences

Multiple adolescent adversities were examined in the “Life as a 16 + Teenager” questionnaire. Assessed were parental divorce/separation, if the teenager experienced severe injuries/illness themselves, if they had academic problems, became a young parent, or experienced bullying or death of a close contact (parent, sibling, close friend). In the “It’s all About You” questionnaire at age 20, young people indicated whether they had housing issues. Other adversities were assessed using the “Teen focus” scale at 17.5 years. Participants rated the experience of conflicts with their parents, suspension from school/college, the number of their close friends, and if they had trouble with the police or lost their job. The “Life at 22+” scale examined abuse in romantic relationships. Additionally, two questionnaires answered by the mother when their child was 13 were used. In the questionnaire “My Teenage Son/Daughter”, mothers rated the loneliness of their teenagers. In the “Well-being of my Teenage Son/Daughter” questionnaire, they indicated whether the adolescent had been involved in an exceptionally stressful situation (accident, abuse, other disasters). 61% of participants experienced no adversities included in our study in the sample. 17% experienced one adversity, 9% experienced two adversities, 5% experienced three adversities, and 8% experienced four or more adversities.

### Mental Health and Wellbeing in Early Adulthood

Mental health at age 22 was examined using the “Life at 22+” questionnaire. Participants indicated whether they had been diagnosed with bipolar disorder, depression, chronic fatigue syndrome, or alcohol use disorder. Lastly, the number of illicit drugs used was surveyed. Well-being factors were assessed using the “Me at 23+” questionnaire. Prevalence rates of all measures can be found in Supplementary Table 2. Please note that the study website contains details of all the available data through a fully searchable data dictionary and variable search tool.

## Data Preparation and Analysis

Data were analysed in R version 4.0.4. (R Core Team, 2021) with R studio version 1.4.1106 (RStudio Team, 2021). Where applicable, we reverse-coded items so that higher scores reflected more negative outcomes. The only items that were coded so that a higher score reflects a more positive outcome are the well-being items and the number of friends item.

### Network Analysis

We conducted two network analyses. The first network captured adversity in childhood and included the ten classic adverse childhood experiences (ACEs, age 1–11), five mental health issues, and two well-being factors in early adulthood (age 22 to 23+). The second network captured adolescent adverse experiences (AAEs). It included the same ten classic ACEs, an additional thirteen AAEs (age 11 to 23+), as well as five mental health issues and two well-being factors in early adulthood (age 22 to 23+). We also conducted a third, exploratory, expanded ACEs network analysis. This expanded network was estimated for childhood but contained eight additional adversities also modelled in the AAE network to enable a more direct comparison of networks in childhood and adolescence (results and methods details are included in the Supplementary Materials).

We used the R package bootnet (*Bootnet Function - RDocumentation*, 2021) to estimate networks based on the Gaussian graphical model (GGM). In network analysis, variables are called nodes and are visualised as circles. Each node represents an adversity or mental health/well-being outcome in our study. The relationships between the nodes are called edges and are visualised as lines between the nodes. Edges in the networks can be interpreted as partial correlation coefficients (Epskamp et al., 2018; Lauritzen, 1996). Since sometimes regularised and sometimes unregularised partial correlations describe the network structure best (Isvoranu & Epskamp, 2021), we used the ggmModSelect method, which estimates Gaussian Graphical Models (GGMs) via stepwise model selection (*EstimateNetwork Function - RDocumentation*, 2021). For regularisation, it uses the LASSO, i.e. the Least Absolute Shrinking and Selection Operator, which prunes out the non-robust edges, resulting in a sparser network (Epskamp et al., 2018; Tibshirani, 1996). The extended Bayesian information criterion (EBIC) is used as a model selection criterion (Chen & Chen, 2008). The ggmModSelect algorithm runs graphical LASSO to obtain 100 models, refits those without regularisation, chooses the best fitting model according to EBIC and then tests those models with stepwise edge changes until there is no improvement of the EBIC (*GgmModSelect Function*, 2021). The results of estimating the networks were plotted using the “qgraph” package (Epskamp et al., 2012) and visualised colour-blind-friendly colormap “viridis” (Garnier et al., 2021). In the network analysis, we have retained all participants who responded to any of the questionnaires at least once. To handle missing data, we have estimated the relationships between adversities, as well as the relationships between adversities and mental health based on pairwise deletion, currently a standard method of handling missing data in network analysis (e.g., see Fried et al., 2018; Epskamp, 2017).

We also conducted a sensitivity check to compare ggmModSelect to EBICglasso as an alternative model selection method (Chen & Chen, 2008). The EBICglasso method, by default, sets the hyperparameter gamma to 0.5. In contrast, the ggmModSelect method, by default, sets the hyperparameter gamma to 0 (*GgmModSelect Function*, 2021). The results of this complementary analysis can be seen in Supplementary Figs. 1–4.

### Clustering – Exploratory Graph Analysis

Clustering was used to identify nodes that are highly interconnected with one another in the network (Hevey, 2018). We used an Exploratory Graph Analysis (EGA, *Hfgolino/EGA*, 2021) to establish whether adversities formed distinct clusters in childhood versus adolescence. EGA uses Walktrap, a weighted network community detection algorithm. Walktrap was used to detect the number of dense subclusters in the partial correlation matrices underlying the network graphs. EGA allowed us to explore which adversities are most likely to co-occur (Golino & Epskamp, 2017; *Hfgolino/EGA*, 2021) during childhood and adolescence.

### Centrality Indices

We calculated centrality indices such as *node strength, closeness, betweenness*, and *expected influence* but have predominantly focused on *node strength*, which aligned most closely with our research interest. Node strength reflects how a node is directly connected with the other nodes in the network. To determine node strength, the number of direct connections to other nodes as well as the absolute strength-values of these connections are taken into account (absolute strength as in that the sign of the edge is not taken into account; Hevey 2018).

### Network Accuracy and Stability

We estimated the reliability of our inferences by calculating the *edge-weight accuracy* and the *node strength stability* using bootnet. Evaluating *edge-weight accuracy* can be achieved by calculating the edge weights in N randomly-allocated bootstrap samples, in our case 2000 (Efron, 1979; Epskamp et al., 2018), based on which confidence intervals can be calculated for the edge weights. Inspecting *node strength stability* allowed us to determine if the order of node strength coefficients remained the same after dropping participants from the sample. The *centrality stability coefficient* (*CS*-coefficient) was inspected to estimate the maximum number of participants that could be dropped while retaining a correlation of ≥ 0.7 between the original node strength indices and the subset samples with dropped participants with a 95% probability (Epskamp et al., 2018). The results of these analyses can be seen in Supplementary Figs. 5 and 6.

### Additional Analyses

We calculated bridge centrality using *bridge strength*. Bridge strength refers to the sum of the absolute value of all edges between a node of a cluster to all the nodes of the opposing cluster (Jones, 2017; Jones et al., 2021; Vanzhula, 2017). Bridge nodes show which specific key nodes connect adversity and mental health clusters and are especially relevant to mental health issues.

We conducted a *network comparison* between the sixteen classic adversities that were assessed both for childhood (age 1–11, “ACEs” network) and adolescence (age 11–23, “AAEs” network). To this end, we used the *Network Comparison Test (NCT)*, a permutation test (van Borkulo, 2018). The *NCT* estimates the network structure and calculates a metric that functions as the observed test statistic. Group membership is multiple times rearranged via permutation, followed by a recalculation of the network structure and test statistic. This results in a reference distribution. The *NCT* then compares the first observed test statistic with this reference distribution, indicating whether the observed test statistic is significantly different (van Borkulo et al., 2017). The test statistics used to compare the two networks are set out to test invariance regarding (1) the global network structure, (2) the edge strength, and (3) the global network strength. The global network structure estimate allowed us to assess whether the structure of the two networks can be considered invariant. If this test statistic is significant, we test the invariance of specific edges in the networks using the edge strength estimate. The global network strength test estimates whether the overall level of connectivity strength was the same across the two networks (Borkulo et al., 2017).

Finally, we examined the relationship between adversities and early adulthood mental health. We assessed the *direct pathways* between adversities and the two mental health nodes using the highest bridge centrality determined in the previous analysis. We additionally computed path diagrams in childhood and adolescence based on the shortest pathway analysis (Brandes, 2008). The path diagram visualises the strongest connections between a respective adversity and the mental health node of interest (regardless of whether the pathway is direct or indirect). If an adversity node does not have a direct edge with the mental health node, the shortest pathway determines the strongest indirect connections via co-occurring adversities (Fritz et al., 2019; Isvoranu et al., 2020). The network analysis code can be accessed here: https://networksofadversities.netlify.app/.

## Results

This study investigates the co-occurrence of adverse experiences in childhood and adolescence and evaluates the link between adversities and adult mental health. We conducted two network analyses to compare the interrelations of childhood and adolescent adversities and their relationships to early adulthood mental health and well-being. First, we determined whether adversity clusters could be detected. Second, we assessed node strength centrality in the childhood and adolescence networks. Third, we calculated which nodes function as “bridges” connecting adversity clusters with mental health issues. Fourth, we used a network comparison test to determine whether classic adversities and mental health networks differ between childhood and adolescence. Finally, we analysed the direct connections of the most central mental health outcomes with the adversities in both age groups using path diagrams.

## The ACEs Network

**Fig. 1:**
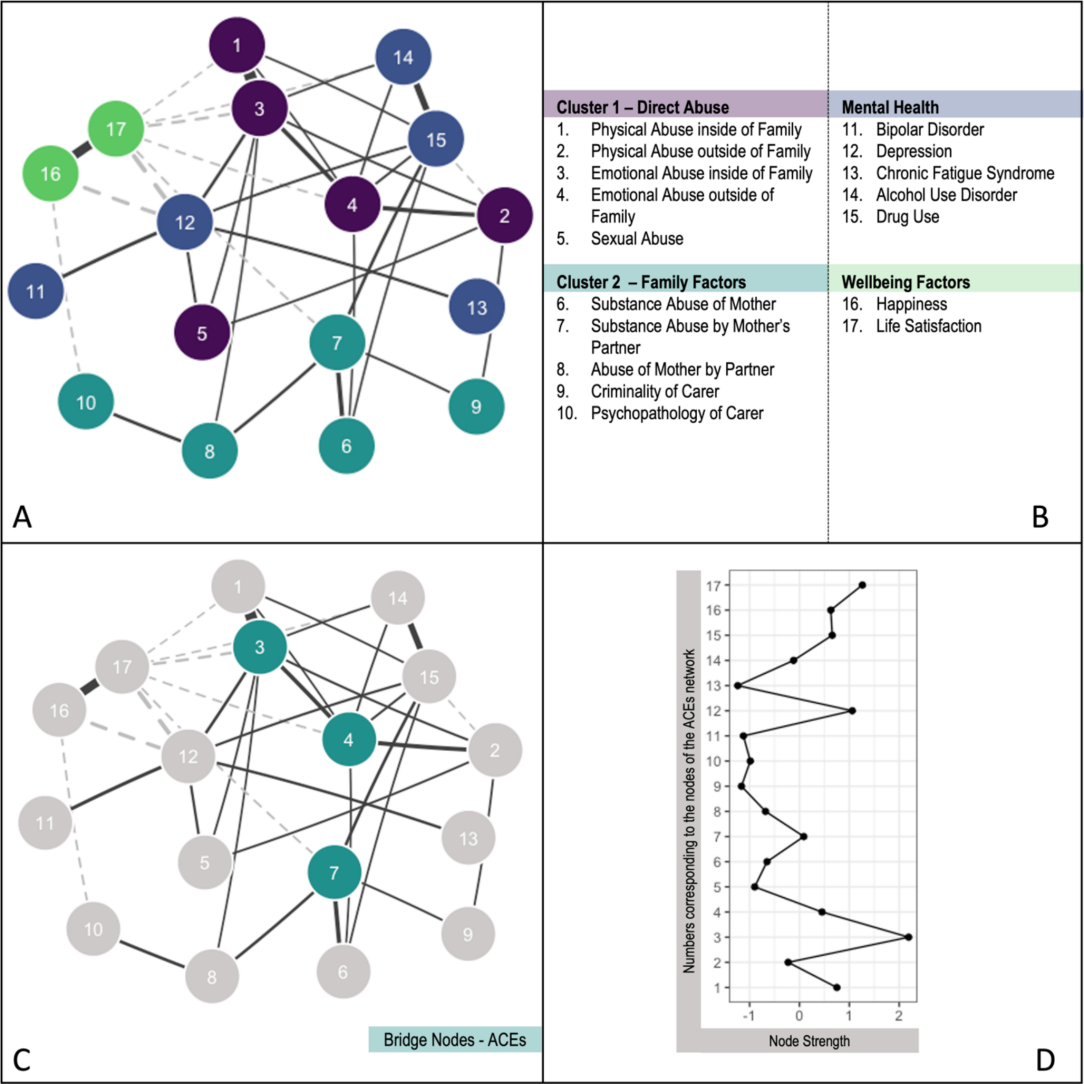
A: The ACE network including two clusters of adversities. Dashed lines represent negative partial correlations, while dark grey lines indicate positive partial correlations. The more saturated the edge, the stronger the partial correlation. B: Network Node Labels. C: Bridge nodes of the adversity cluster (dark green). D: Strength scores of the ACEs nodes. Strength refers to the node strength of the ACEs network. Standardised Z-scores are shown for node strength (see B). For interpretation of the references to colour in this figure legend, please refer to the web version of this article.

We found two clusters in the ACEs network. Cluster 1 included mental, physical, and sexual abuse (direct abuse cluster, Fig. 1). Cluster 2 contained variables related to the family environment, such as parental substance abuse and parental partner cruelty (family factors cluster, Fig. 1).

We assessed the relationships between ACEs, mental health, and well-being by estimating the node strength. Closeness, betweenness, and expected influence indices are included in Supplementary Fig. 7. Emotional abuse inside the family (node 3), physical abuse inside the family (node 1), and emotional abuse outside the family (node 4) showed the highest node strength. It can therefore be considered particularly central to the ACE network. Emotional abuse inside the family refers to an adult inside the family shouting at or insulting the child. In contrast, emotional abuse outside the family refers to an adult outside the family shouting at or insulting the child. Life satisfaction (node 17) and depression (node 12) showed the highest node strength of the mental health and well-being variables. See Fig. 1.

Bridge nodes have the highest number of connections with the opposing cluster – i.e., they function as “bridges” connecting adversities to mental health. Bridge nodes of the ACEs were emotional abuse inside the family (node 3), emotional abuse outside the family (node 4), and substance abuse by the mother’s partner (node 7). Emotional abuse inside the family was most strongly associated with depression (node 12, *r* = .10) and life satisfaction (node 17, *r* = − .09). Emotional abuse outside the family was most strongly correlated with drug use (node 15, *r* = .06) and life satisfaction (node 17, *r* = − .06). Substance abuse by the mother’s partner was most strongly related to drug use (node 15, *r* = .10) and life satisfaction (node 17, *r* = − .06). See Fig. 1.

In summary, the findings for the ACE networks highlight that mental and physical abuse are central to networks of childhood adversities. Especially relevant here was emotional abuse since it was not only central but also functioned as a bridge node connecting the adversity to the mental health cluster.

## The AAEs Network

**Fig. 2:**
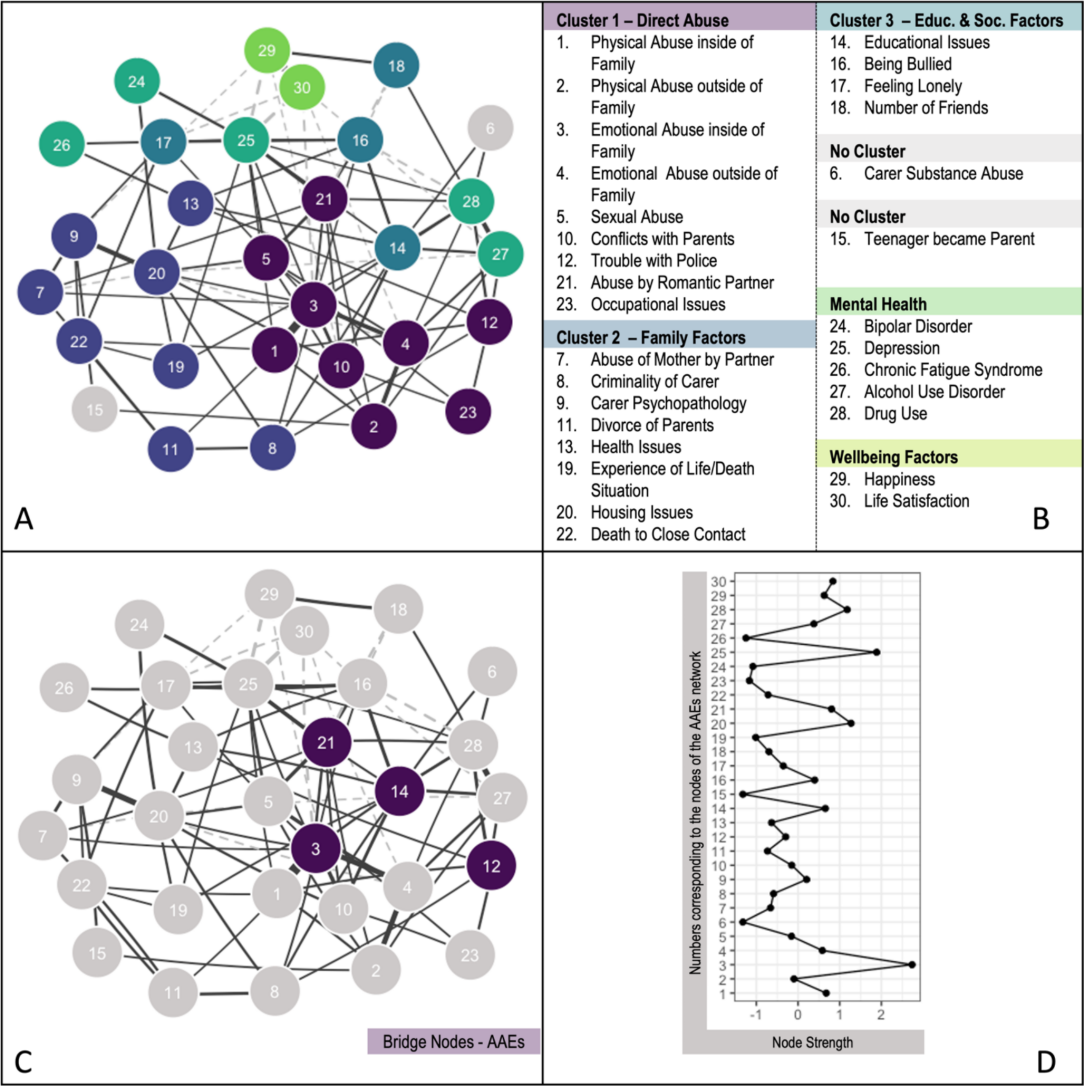
A: The AAEs network including the clusters of adversities. Dashed lines represent negative partial correlations, while dark grey lines indicate positive partial correlations. The more saturated the edge, the higher the correlation. B: Network Node Labels. C: Bridge nodes of the adversity cluster (dark lilac). D: Strength scores of the AAEs nodes. Strength refers to the node strength of the AAEs network. Standardised Z-scores are shown for node strength (see B). For interpretation of the references to colour in this figure legend, please refer to the web version of this article.

We found three clusters for the AAEs network. Cluster 1 included adversities that directly impact adolescents (direct abuse cluster, Fig. 2). This cluster contained the same adversities as in the ACEs cluster: emotional abuse, physical abuse, and sexual abuse. Additional adversities in cluster 1, not included in the ACE network, were conflicts with parents, trouble with the police, abuse by a romantic partner, and occupational issues. Cluster 2 mainly contained indirect abuse through family factors (family factors cluster, Fig. 2). It had similar variables as the ACEs network, e.g., parental partner cruelty, parent criminality, and parental psychopathology. It included additional variables, however: parental divorce, health issues, the experience of life/death situation, housing issues, and death of close contact. A third cluster contained variables related to school and social life, specifically educational issues, being bullied, feeling lonely, and the number of friends (educational and social factors cluster).

We assessed the relationship between AAEs, mental health and well-being by inspecting the node strength between variables. Closeness, betweenness, and expected influence indices are included in Supplementary Fig. 8. Emotional abuse inside the family (node 3), housing issues (node 20), abuse by a romantic partner (node 21), physical abuse inside the family (node 1), and educational issues (node 14) showed the highest node strength of the adversity variables, indicating that these adversities were particularly central to the network. Depression (node 25) and drug use (node 28) showed the highest strengths in the mental health and well-being variables. See Fig. 2.

Bridge nodes of the AAE clusters were, in declining importance: educational issues (node 14), abuse by romantic partner (node 21), emotional abuse inside the family (node 3), and trouble with the police (node 12). Educational issues were most strongly related to alcohol use (node 27, *r* = .11) and drug use (node 28, *r* = .09). Abuse by romantic partner was most strongly related to depression (node 25, *r* = .15) and drug use (node 28, *r* = .07). Emotional abuse inside the family was most strongly related to life satisfaction (node 30, *r* = − .08) and drug use (node 28, *r* = .08). Lastly, trouble with police was most strongly related to drug use (node 28, *r* = .12) and alcohol use (node 27, *r* = .09). See Fig. 2. Partial correlation coefficients were slightly higher for AAEs compared to ACEs. This might reflect a shorter time gap between experiencing these adversities and the assessment of mental health disorders.

## Network Comparison and the Expanded ACEs Network

The network comparison included the nine classic ACEs in childhood and adolescence as well as the mental health and well-being nodes. The AAEs not included in the ACEs were not part of the network comparison since it is only possible to compare networks with the same nodes. The network invariance test showed that the difference between the network structures was not significant (*M* = 0.092, *p* = .248). Since the network structure did not show significant differences, we did not test specific edge differences in the networks. The global strength invariance test was also not significant (*S* = 0.243, *p* = .142). Overall, we found no evidence for a difference between the networks of classic adversities in childhood and adolescence, indicating that the networks of classic adversities in both age groups are similar. See Supplementary Fig. 9.

The clusters in the expanded ACEs Network remained similar, with a new cluster containing most of the additional items (e.g., educational issues). Again, emotional abuse inside the family showed the highest adversity node strength, and depression showed the highest mental health node strength. For more information, see Supplementary Fig. 11.

## Direct Pathways

**Fig. 3:**
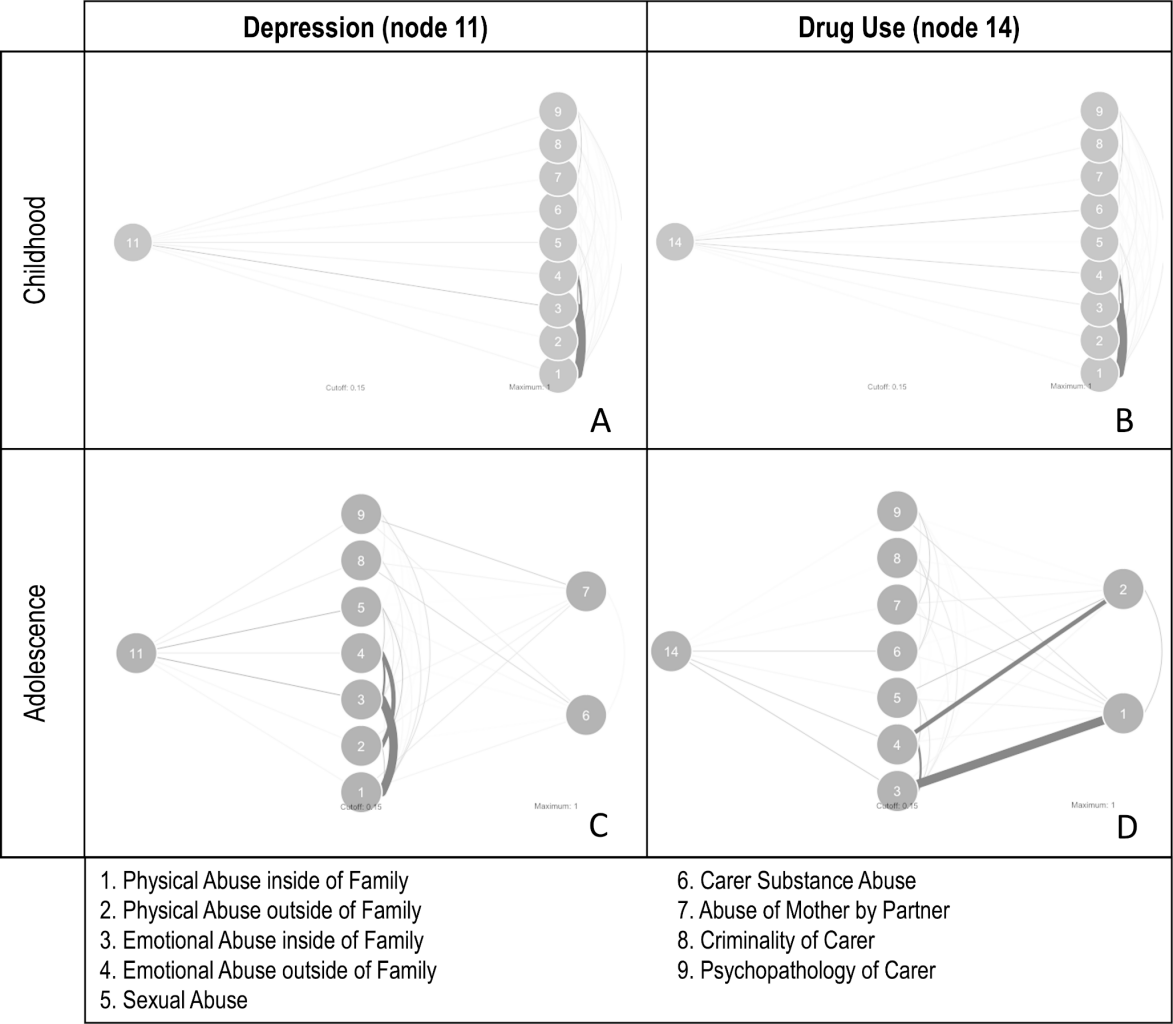
Depression (A and C) and drug use (B and D) pathways to adversities in childhood (A and B) and adolescence (C and D). The more saturated the edge, the stronger the association

To further examine differences in the networks, we investigated the link between adversities in childhood and adolescence and early adulthood mental health issues. For this, we examined the two mental health nodes with the highest node centrality, drug use (node 11) and depression (node 14). The most notable difference between the age groups was that all childhood adversities were directly connected to mental health issues in young adulthood, whilst several adversities during adolescence had indirect connections with mental health in young adulthood. For example, parental substance abuse (node 6) and parental partner cruelty (node 7) showed an indirect connection to depression via multiple other adversities (e.g., criminality or psychopathology of carer). Similarly, physical abuse inside the family (node 1) and physical abuse outside the family (node 2) were indirectly connected with drug use, e.g., most strongly through emotional abuse inside and outside the family environment. See Fig. 3. This suggests that adversities have different effects on mental health issues in later life, depending on the age at which they are experienced.

In summary, we conducted network analysis, and we determined firstly which adversities cluster together, secondly the centrality (i.e., the highest node strength) of adversities in both age groups and thirdly, which nodes connect the adversity cluster with the mental health cluster (i.e., bridge strength). Using these methods, we found that hypothesis (1) was partially supported: While we did find two similar clusters (direct abuse and family factors), we found an additional cluster in adolescence. This additional cluster mainly comprised adversities not included in the childhood network (e.g., social and educational factors). Hypothesis (2) was partially supported. We found both similarities (e.g., emotional abuse is one of the most relevant adversities at both ages) and differences (e.g., additional adversities such as educational issues become relevant during adolescence) in the networks of adversities in both age groups. In childhood, direct traumatisation in the family environment was especially detrimental, while during adolescence, direct abuse in the family and social/educational environment became important. In line with Hypothesis (3), we found a correlation between the classic adversities in childhood and adolescence and mental health outcomes in early adulthood. In both age groups, depression appears to be the most strongly associated mental health disorder. Hypothesis (4) was also supported. While we cannot directly compare the networks for childhood containing the classic ACEs with the adolescence network, we found a correlation between the non-ACEs adversities in adolescence and mental health outcomes in early adulthood.

We conducted two additional exploratory analyses to further scrutinise the differences between the childhood and adolescence networks. First, we compared the ACEs and AAEs network structures of classic adversities and mental health. The structure of networks in both age groups did not significantly differ. Second, we analysed the direct connections of the most central mental health outcomes with the adversities in both age groups. The results indicated that childhood adversities were directly connected to mental health issues, whilst several adversities during adolescence showed indirect connections with mental health in young adulthood.

## Discussion

This study aimed to investigate the co-occurrence of adverse experiences in childhood and adolescence and their influence on early adulthood mental health. To investigate this, we conducted network analyses to examine the relationships of adversities among each other and their correlation to mental health disorders. We determined adversity clusters, assessed node strength centrality in the childhood and adolescence networks and examined which nodes function as “bridges” to connect adversity clusters with mental health issues. Lastly, we compared the networks of classic adversities in childhood and adolescence and their relationship to mental health outcomes. Our results showed similarities (e.g., emotional abuse as the most important adversity) and differences (e.g., educational issues became more relevant during adolescence) in the child and adolescence networks. While it is not possible to directly compare both networks, the results highlight that there may be a difference between influential adversities and clusters depending on age. The results also showed a connection between adversities and mental health outcomes in later life, with depression as a particularly central outcome of adverse experiences. This study highlights the utility of network analysis for adversity research. Not only can the clustering of adversities be examined, but individual adverse experiences and their connections to later life outcomes can also be illustrated. Importantly, our pathway analysis showed that depending on the age at which adversities are experienced, adversities might have different effects on mental health issues in early adulthood.

## Adversity Clusters in Childhood

We hypothesised (1) that there would be distinct adversity clusters during childhood. In childhood, we found two clusters in the network of adversity: a direct abuse and a family factors cluster. The direct abuse cluster included physical, mental, and sexual abuse. The family factors cluster included the other classic adversities in childhood, which exert more indirect influences on the child’s life, such as parental substance abuse and criminality of parents or carers. This dovetails with Breuer et al., (2020), who also found two clusters based on a clinical inpatient sample: Direct traumatisation through neglect and indirect traumatisation through adverse experiences such as abuse of the mother by her partner. Our study extends previous work by highlighting that a similar pattern holds in the general population and by showing that the direct abuse cluster appears especially detrimental. This indicates that direct abuse could be a promising target for future intervention research.

In adolescence, we found three clusters of adversity. Two clusters were similar to those in childhood (direct abuse and family factors). The direct abuse cluster comprised the adversities also found in this cluster in childhood, with additional items, such as conflicts with parents, abuse by a romantic partner and trouble with the police. The family factors cluster contained almost all variables seen in the childhood cluster with additional items such as parents’ divorce or housing issues. The third cluster contained additional educational and social factors that were mostly only assessed during adolescence and included educational issues and peer relationship factors such as bullying, loneliness, and the number of friends. This highlights those additional adversities, reflecting the age-typical environment of adolescents, which may need to be screened for in youth.

Previous research proposed a dimensional model of adversity and psychopathology, in which threat (e.g. the experience of harm or threat of harm) and deprivation (e.g. the absence of expected environmental inputs) function as different dimensions of adversities with different mechanisms and impacts on developmental pathways (McLaughlin et al., 2014; McLaughlin & Sheridan, 2016; Miller et al., 2018). Using network analysis, a study by Sheridan et al., (2020) found support for this dimensional approach. We did not include classic deprivation items in this study as we did not have relevant measures. However, the direct abuse cluster speaks to threat, as physical and emotional harm was included here. The social and educational cluster comprised items such as a lack of educational affirmation and social approval, which speak to deprivation. Therefore, this study could indicate support for a dimensional model of adversity and their developmental influences. That said, the social and educational cluster also contained bullying, which would be classed as a threat rather than deprivation. Thus, further research needs to be conducted to support the dimensional model of adversity and psychopathology.

## Influential Adversities in Childhood

We hypothesised (2) that there would be distinct influential adversities in childhood and adolescence. In the childhood network, emotional abuse inside the family, physical abuse inside the family, and emotional abuse outside the family were most central. Mental and physical abuse were also key nodes in the childhood adversity network. They functioned as bridge nodes between the direct abuse cluster and the mental health and well-being factors cluster. Emotional abuse includes being shouted at, verbally insulted, and age-inappropriate punishments, while physical abuse includes abuse such as being hit, smacked, or punched. Until recently, there has been a common belief that certain types of mistreatment, such as physical and sexual abuse, are more detrimental than other forms, such as emotional abuse and neglect (Vachon et al., 2015). Our study highlights emotional abuse as a central and consequential form of abuse that may be important to the development of mental health problems, such as depression, during later life. This is in line with Spinazzola et al.‘s (2014) study, which demonstrated that psychologically maltreated youth showed similar or more severe outcomes in terms of disorders, symptoms and behavioural issues compared to sexually and physically abused children. We calculated the number of participants who experienced the classic adversities exclusively in childhood or adolescence and how many experienced it in both age groups (see Supplementary Fig. 10). In this study, emotional abuse was the most common type of abuse and was often experienced by the same individual in both age groups. Critically, emotional abuse is rarely the focus of current interventions for young people despite its widespread occurrence and potential large negative influence on lifespan development (Spinazzola et al., 2014).

In the adolescence network, emotional abuse inside the family, housing issues, mistreatment by a romantic partner, and physical abuse inside the family had the highest node strength in the network overall, highlighting the continued importance of direct abuse for mental health outcomes. However, the bridge nodes connecting the clusters to the mental health and well-being items differed from those in childhood. Most of the bridge or key nodes were part of the educational and social factors cluster in adolescence. Bridge nodes here included educational issues (such as doing badly at schoolwork), romantic partner abuse, emotional abuse inside the family, and trouble with the police. Therefore, this study highlights emotional abuse as one of the central forms of abuse that needs to be addressed independently of age.

Educational issues in adolescence showed the strongest connection to mental health and well-being in adulthood. This is in line with a study by DeAngelis & Dills (2020), which found that the educational environment, including the choice of schools, influenced the likelihood of mental health issues in adulthood. A possible mechanism may be that adolescents with better educational outcomes have a sense of control of their lives or better occupational opportunities, enabling better life chances and promoting health in adulthood (Mirowsky & Ross, 2017). Adolescents’ experiences in school can have lasting consequences and appear to be especially correlated with mental health issues later. This study, therefore, underlines the importance of addressing educational issues in adolescence.

## The Connection Between Adversities and Mental Health

We hypothesised (3) that there will be a relationship between classic adversities in childhood and adolescence and mental health outcomes, and (4) we expect that non-ACE adversities in adolescence will be related to mental health. In line with these hypotheses, we found a partial correlation between early adulthood mental health and adversities experienced in childhood and adolescence. Depression, drug use and lower life satisfaction were common outcomes in both age groups. This result is similar to other studies (De Venter et al., 2013; Felitti et al., 2019; Schilling et al., 2007), highlighting the potential impact of adversities experienced in childhood and adolescence on mental health and well-being. Pathway analysis further showed that there might be a change over time in the effect of specific adversities on mental health, with effects becoming more indirect in adolescence.

## The Expanded ACEs Network

We conducted an additional exploratory analysis to expand the ACEs network and include a broader range of adverse experiences in childhood. This expanded childhood network contained the classic ACEs, and eight additional adversities matched to the AAE network to enable a more direct comparison of networks in childhood and adolescence. The expanded ACEs network showed similarities with the original ACEs network. The nodes with the highest node strengths were robust across analyses, with emotional and physical abuse, in particular, being central to the network. The expanded ACEs network, therefore, highlights abuse in the family again as a central form of abuse, despite the inclusion of other potential forms of ACEs. The most pronounced difference was in the bridge nodes. The death of a family member and admission to the hospital became relevant in addition to the emotional abuse item seen in the original ACEs network. This underlines the importance of expanding the current ACEs framework further to facilitate a broader understanding of potential adversities during childhood and their long-term consequences (e.g., Cronholm et al., 2015; Karatekin & Hill, 2018; White et al., 2019).

## Strengths and Limitations

The strength of this study is the use of a large cohort with a longitudinal, partially prospective design. We included a wide range of adversities in two age groups, highlighting their interconnectedness using a complex systems approach. There are several limitations to the study. This includes that it is not possible to directly compare childhood and adolescence networks since the adolescence networks had additional adversities that were not included in the younger age group due to limited data availability. Also, it is not possible to infer causality in network analysis since the network is based on correlations. Hence, future studies should consider using more complex study designs to determine the causality of adversities on mental health issues. Next, some of the adversity scales used here were retrospective measures, which might lead to limited recall or recall biases (Talari & Goyal, 2020). Also, parental substance abuse during adolescence included only maternal substance abuse, but during childhood, the mother’s partner’s substance abuse was found to be especially relevant. Moreover, we included only a small number of possible mental health outcomes due to availability. Anxiety, in particular, will be important to study as an outcome in future research, as anxiety is closely linked to adversity (King, 2021; Raposo et al., 2014). Lastly, due to the longitudinal study design, the data set contains missing data. For example, the response rate for housing issues was – compared to other measures – relatively low (N ≈ 1,200). Future studies should consider using different measurements of the ACEs/AAEs and additional relevant mental health and well-being outcomes.

## Conclusion

We found that adversities divide into direct and indirect traumatisation via family circumstances during childhood. Emotional and physical abuse were central to the network of adversities. Emotional abuse was especially connected to mental health issues in early adulthood, highlighting that preventing this type of adversity may be relevant in addressing depression as a public health issue. During adolescence, adversities can be divided into direct abuse, family, and social/educational factors. Adolescent adversities such as housing issues and abuse by a romantic partner were central in the network of adversities. Educational issues were strongly connected to mental health issues later in life. This has implications for intervention development and mental health policy: Different adversities may need to be considered to support adolescents compared to children. Future research should deepen this understanding and work towards a comprehensive approach to tackle adversity across the lifespan.

### Electronic Supplementary Material

Below is the link to the electronic supplementary material.

Supplementary Material 1
