# Supplementary material for: Networks of Adversity in Childhood and Adolescence and Their Relationship to Adult Mental Health
Source: Res Child Adolesc Psychopathol. 2022 Nov 4;51(12):1769–84. doi: 10.1007/s10802-022-00976-4 (PMC10661796; doi:10.1007/s10802-022-00976-4)
Supplement: Supplementary file 1 — Supplementary Material 1 [file 10802_2022_976_MOESM1_ESM.docx]

**Supplementary Materials**

**Supplementary Tables**

Table 1

*Demographics of the ALSPAC Sample (N =* 14,901*)*

| **ALSPAC participants** |  |
| --- | --- |
| Gender |  |
| Male | 51.2% |
| Female | 48.8% |
| Ethnic Group  White  Other  *Missing* | 77.2%  2.1%  *20.7%* |
| Social class based on maternal occupation  Lowest 33%  Middle 33%  Highest 33%  *Missing* | 15.5%  33.1%  22.6%  *28.9%* |

Supplementary Table 2

*Prevalence rates of childhood and adolescence adversities in the ALSPAC Sample (N = 14.901)*

| Childhood Adversities |  |  | | |  | | | |  | |  |  |  |
| --- | --- | --- | --- | --- | --- | --- | --- | --- | --- | --- | --- | --- | --- |
| Frequency | *Never* | *Rarely* | | | *Sometimes* | | | | *Often* | | *Very often* | ***Total n*** | ***Missing*** |
| Physical Abuse inside Family | 16.9% | 6.9% | | | 0.9% | | | | 0.2% | | 0.1% | 3,912 | 75% |
| Physical Abuse outside Family | 24.9% | 0.2% | | | 0.1% | | | | 0.0% | | 0.0% | 3,943 | 74.8% |
| Mental Abuse inside Family | 9.8% | 12.2% | | | 2.4% | | | | 0.7% | | 0.2% | 3,950 | 74.7% |
| Mental Abuse outside Family | 17.5% | 6.9% | | | 0.7% | | | | 0.1% | | 0.0% | 3,952 | 74.7% |
| Frequency | *No* | *Once* | | | *More than once* | | | | | | *-* |  |  |
| Sexual Abuse | 24.2% | 0.7% | | | 0.2% | | | | - | | - | 3,932 | 74.9% |
| Frequency | *Not at all* | | | *Sometimes* | | *Often* | | | *Everyday* | | |  |  |
| Substance Abuse Mother | 59.2% | | | 5.4% | | 0.0% | | | 0.0% | | - | 10,106 | 35.4% |
| Substance Abuse of Partner | 33.6% | | | 0.8% | | 0.0% | | | 0.0% | | - | 5,389 | 65.6% |
| Frequency | *No* | *Yes* | | | - | | | | - | | - |  |  |
| Parental Partner Cruelty | 54.2% | 7.4% | | | - | | | | - | | - | 9,642 | 38.4% |
| Criminality of Parent (Partner) | 38.9% | 0.9% | | | - | | | | - | | - | 6,223 | 60.2% |
| Psychopathology of Parents | 25% | 0.9% | | | - | | | | - | | - | 6,223 | 60.2% |
| Adolescence Adversities |  |  | | |  | | | |  | |  |  |  |
| Frequency | *Never* | *Rarely* | | | *Sometimes* | | | | *Often* | | *Very often* | ***Total n*** | ***Missing*** |
| Physical Abuse inside Family | 21.2% | 3.1% | | | 0.5% | | | | 0.1% | | 0.1% | 3,912 | 75% |
| Physical Abuse outside Family | 24.5% | 0.4% | | | 0.2% | | | | 0.0% | | 0.0% | 3,935 | 74.8% |
| Mental Abuse inside Family | 11.2% | 11.0% | | | 2.2% | | | | 0.7% | | 0.3% | 3,946 | 74.8% |
| Mental Abuse outside Family | 17% | 6.6% | | | 1.3% | | | | 0.2% | | 0.1% | 3,939 | 74.8% |
| Frequency | *No* | *Once* | | | *More than once* | | | | | | *-* |  |  |
| Sexual Abuse | 23.5% | 1.1% | | | 0.4% | | | | - | | - | 3,923 | 74.9% |
| Frequency | *Never* | *Rarely* | | | *Sometimes* | | | | *Often* | | *Very often* |  |  |
| Abuse by Romantic Partner | 20.9% | 3.2% | | | 0.9% | | | | 0.3% | | 0.2% | 3,997 | 74.4% |
| Frequency | *Not True* | | *Sometimes True* | | | | | *True* | | | - |  |  |
| Loneliness | 38.0% | | 6.6% | |  | | | 0.5% | | | - | 7,072 | 54.8% |
| Frequency | *0* | *1* | | | *2-4* | | | | *5-9* | | *10-20+* |  |  |
| Number of close Friends | 0.1% | 0.5% | | | 8.1% | | | | 11.1% | | 5.5% | 4,115 | 73.7% |
| Frequency | *Not at all* | | | *Sometimes* | | *Often* | | | *Everyday* | | |  |  |
| Maternal Substance Abuse | 24.8% | | | 0.2% | | - | | | - | | - | 3,917 | 75% |
| Frequency | *Never* | *Once* | | | *Twice* | | *Three times* | | | *More than three* | | | |
| Criminality of Teenager | 21.6% | 2.0% | | | 0.7% | | 0.3% | | | 0.7% | | 3,943 | 74.8% |
| Frequency | *No* | *Yes (age 11)* | | | | *Yes (age10/11 & 11)* | | | | | |  |  |
| Parental Partner Cruelty | 44.6% | 1.5% | | | | 0.1% | | |  | |  | 7,231 | 53.8% |
| Frequency | *No* | *Yes* | | | - | | | | - | | - |  |  |
| Criminality of Parents | 31.3% | 1.1% | | | - | | | | - | | - | 5,076 | 67.6% |
| Conflicts with Parents | 23.9% | 5.5% | | | - | | | | - | | - | 4,597 | 70.6% |
| Parental separation/Divorce | 29.7% | 2.8% | | | - | | | | - | | - | 5,088 | 67.5% |
| Serious Illness of Teenager | 28.3% | 4.1% | | | - | | | | - | | - | 5,063 | 67.6% |
| Educational Failure | 15.7% | 5.1% | | | - | | | | - | | - | 3,242 | 79.3% |
| Parenthood of Teenager | 32.2% | 0.1% | | | - | | | | - | | - | 5,059 | 67.6% |
| Bullying | 26.7% | 5.7% | | | - | | | | - | | - | 5,065 | 67.6% |
| Death of close contact | 29.1% | 3.2% | | | - | | | | - | | - | 5,055 | 67.7% |
| Housing Issues | 7.6% | 0.1% | | | - | | | | - | | - | 1,204 | 92.3% |
| Life-Threatening Situation | 37.6% | 7.4% | | | - | | | | - | | - | 7,041 | 55% |
| Unemployment | 28.4% | 1.0% | | | - | | | | - | | - | 4,594 | 70.6% |

**Supplementary Figures**


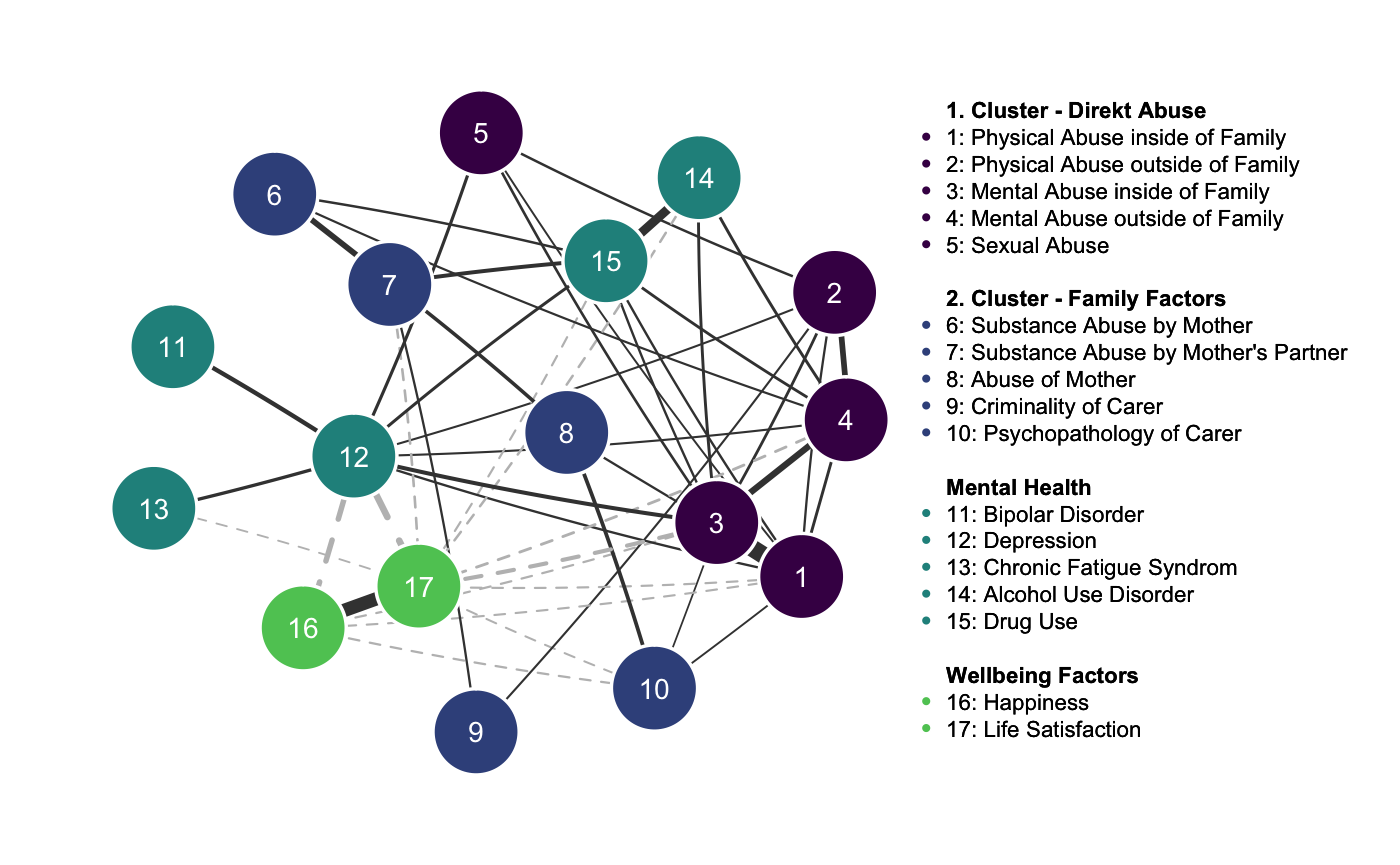


*Supplementary Figure 1*

The ACE network including two clusters of adversities and network labels. Dashed lines represent negative partial correlations, while dark grey lines indicate positive partial correlations. The more saturated the edge, the stronger the partial correlation.


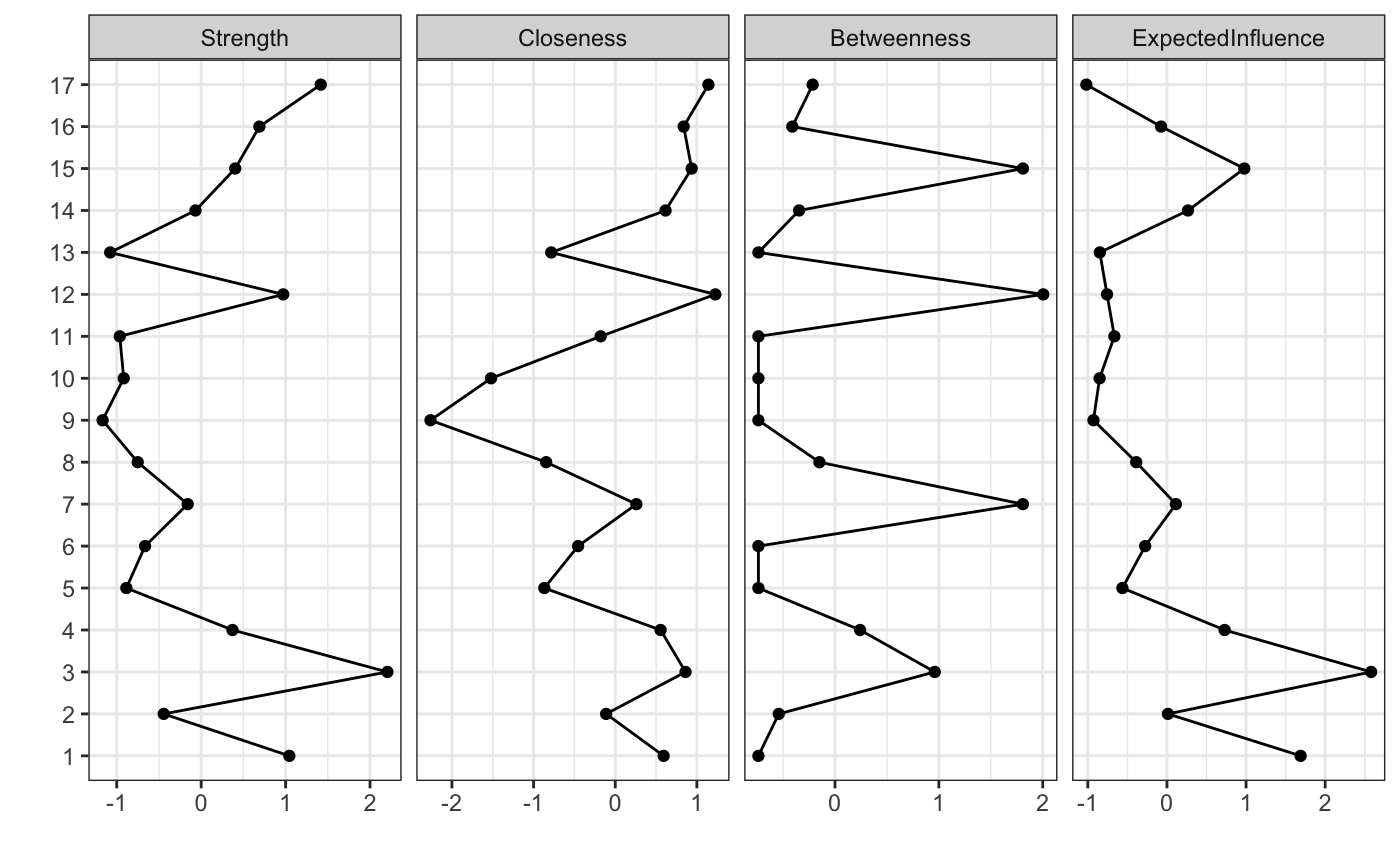


*Supplementary Figure 2*

Node strength. Strength refers to node strength of the ACEs network. Standardised Z-scores with numbers corresponding to the ACEs network.


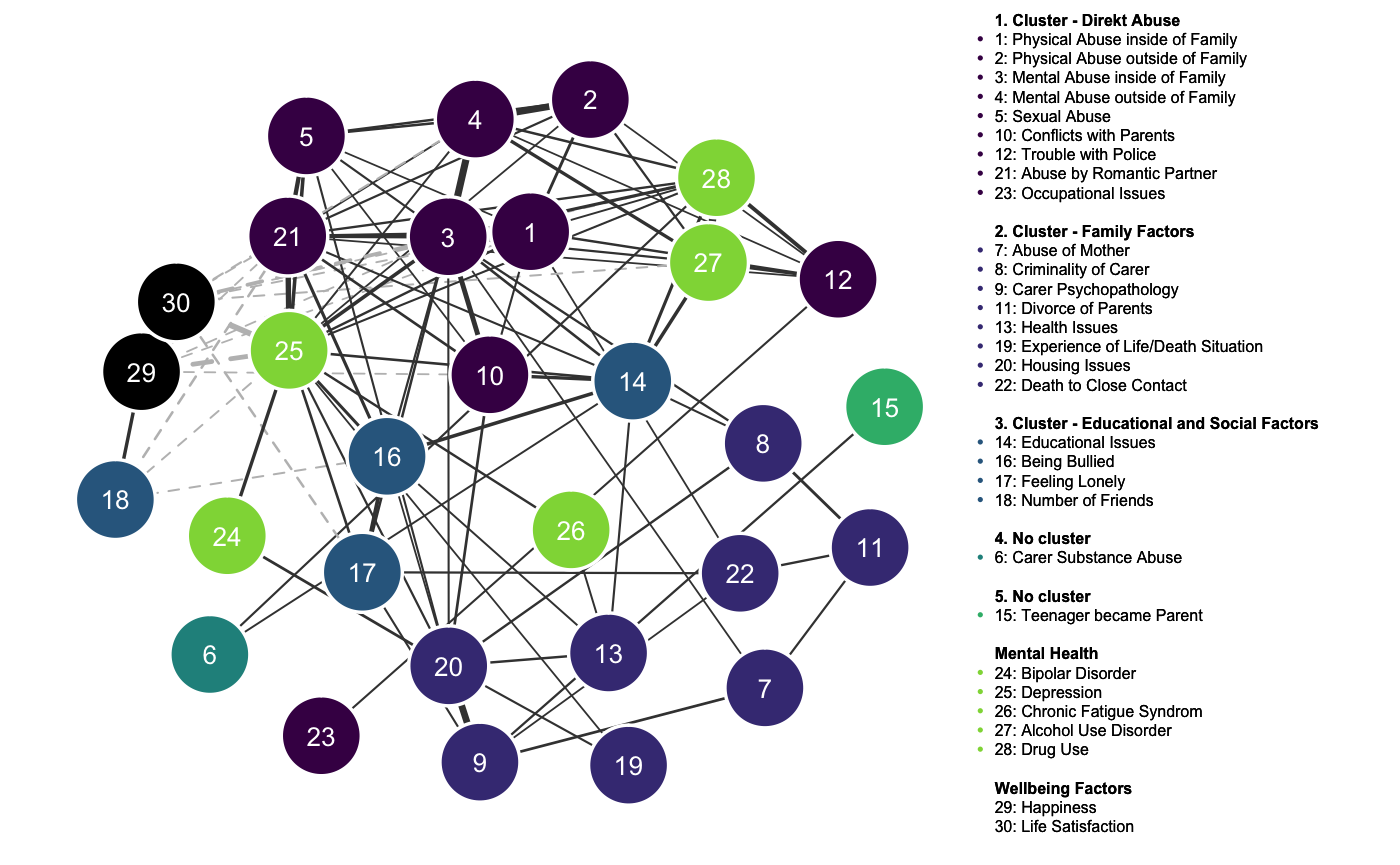


*Supplementary Figure 3*

The AAE network including two clusters of adversities and network labels. Dashed lines represent negative partial correlations, while dark grey lines indicate positive partial correlations. The more saturated the edge, the stronger the partial correlation.


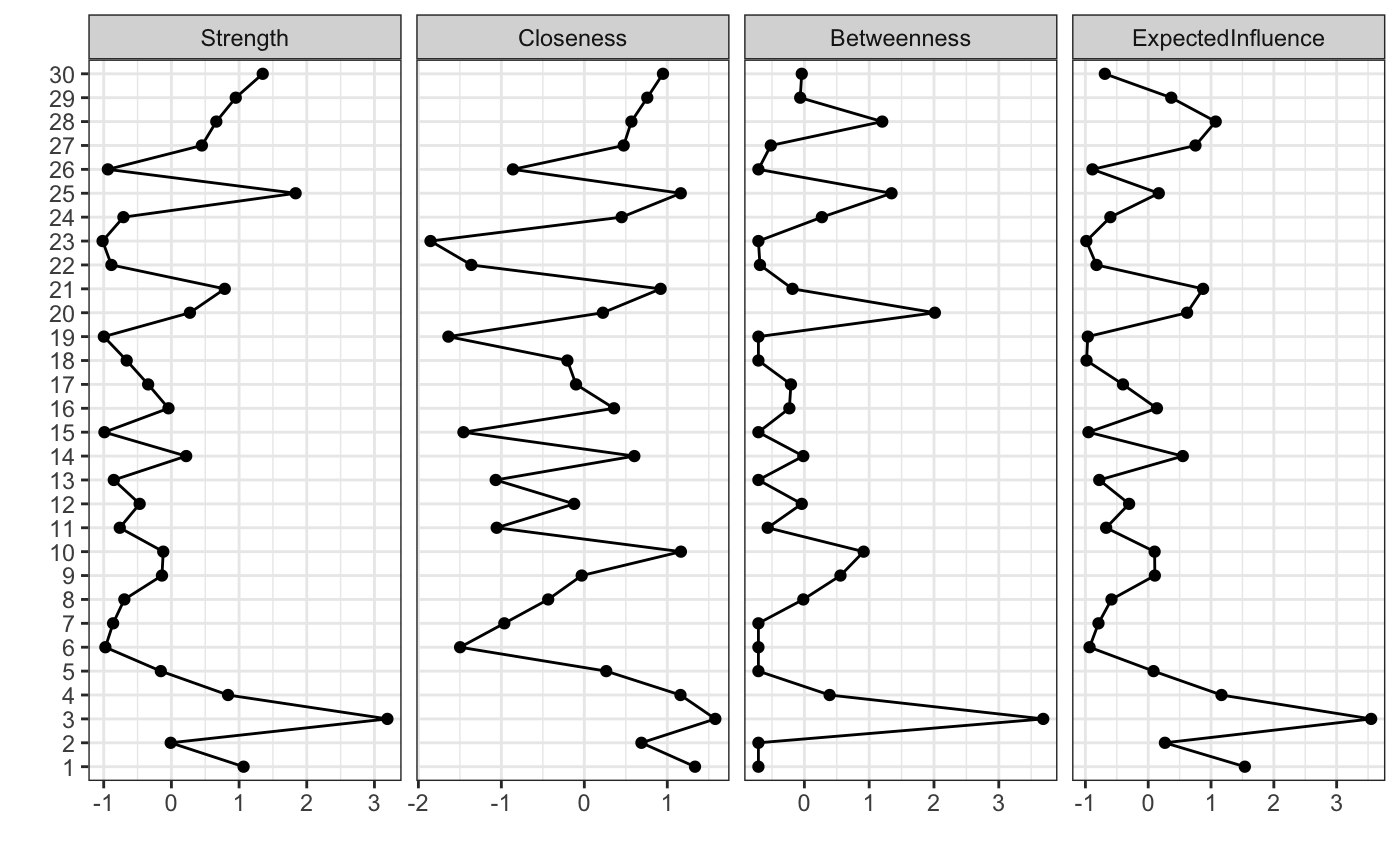


*Supplementary Figure 4*

Node strength. Strength refers to node strength of the AAEs network. Standardised Z-scores with numbers corresponding to the AAEs network.

| 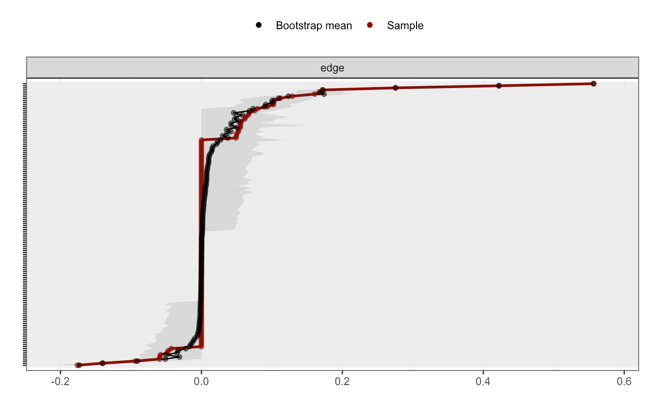 | 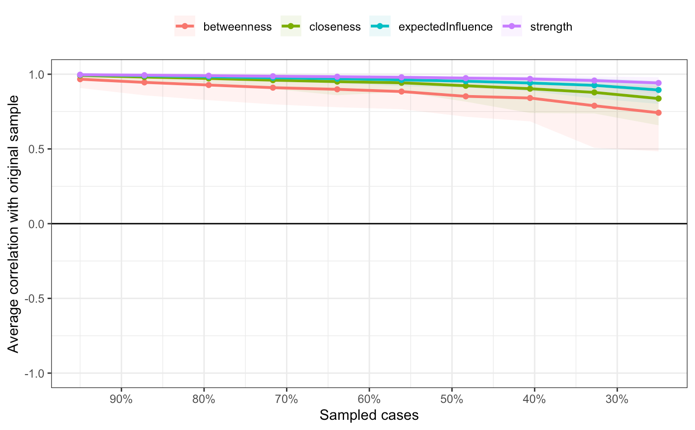 |
| --- | --- |

*Supplementary Figure 5*

Bootstrapping results of the ACEs network. Betweenness: 0.594, Closeness: 0.206, Edge: 0.75, ExpectedInfluence: 0.75, Strength: 0.75

| 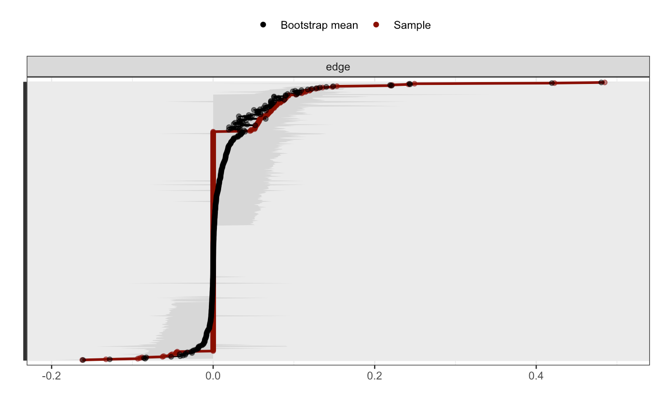 | 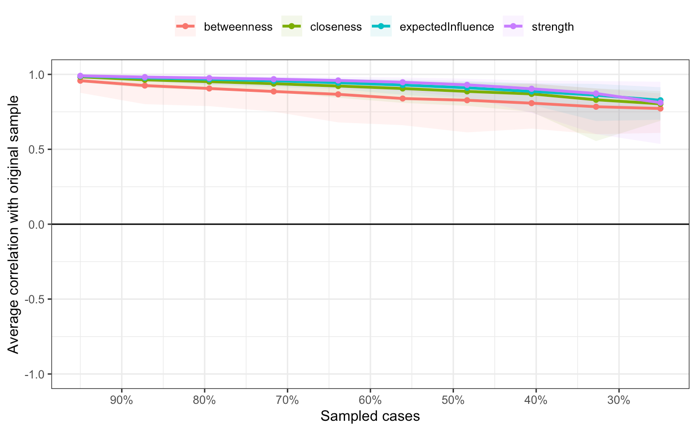 |
| --- | --- |

*Supplementary Figure 6*

Bootstrapping results of the AAEs network. Betweenness: 0.361, Closeness: 0.439, Edge: 0.672, ExpectedInfluence: 0.75, Strength: 0.594


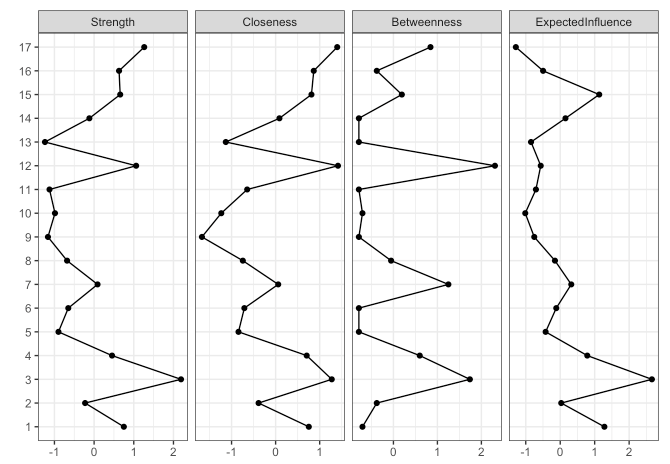


*Supplementary Figure 7*

Node strength. Strength refers to node strength of the ACEs network. Standardised Z-scores with numbers corresponding to the ACEs network.

*
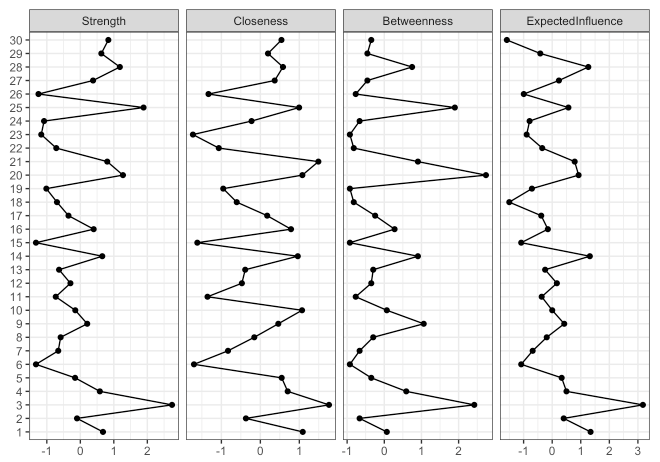
*

*Supplementary Figure 8*

Node strength. Strength refers to node strength of the AAEs network. Standardised Z-scores with numbers corresponding to the AAEs network.

*
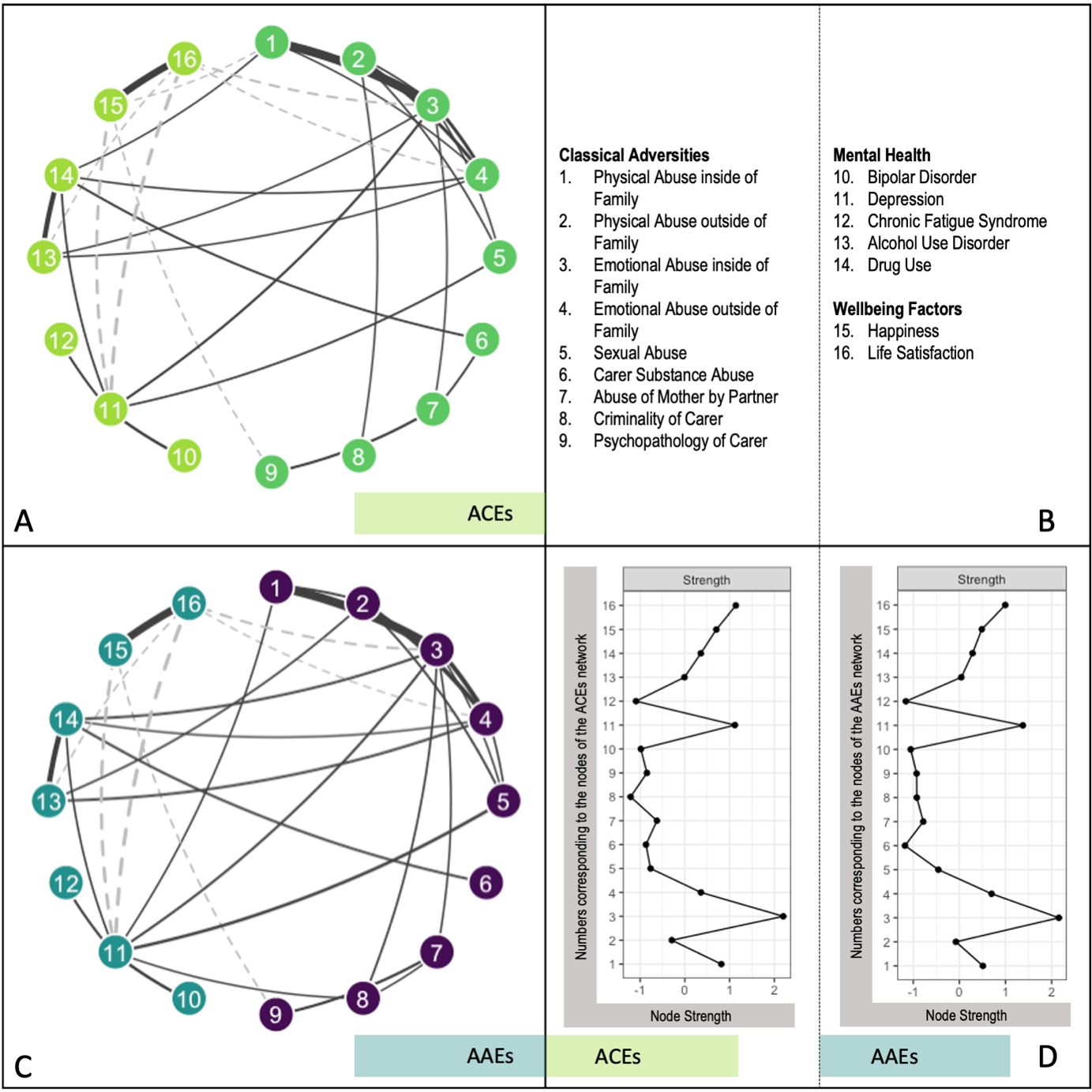
*

*Supplementary Figure 9*

A: The ACEs network of adversities, including mental health and well-being factors. Dashed lines represent negative correlations, while dark grey lines indicate positive partial correlations. The more saturated the edge, the higher the correlation. B: Node label of the networks. C: The AAEs network of adversities and mental health. D: Node strength of ACEs (left) compared to AAEs (right) including mental health and well-being factors. Standardised Z-scores with numbers corresponding to the network below.

*Supplementary Figure 10*

The number of participants experiencing each of the classical nine adversities per age group or in both age groups. Psychopathology was not included since it was not differentiated between childhood and adolescence.

**Supplementary Analysis**

**The expanded ACEs network**

We conducted an additional exploratory analysis to expand the ACEs network and include a broader range of adverse experiences in childhood. This expanded childhood network contained the classic ACEs, and eight additional adversities were modelled in the AAE network to enable a more direct comparison of networks in childhood and adolescence.

**Methods**

**Measures**

In addition to the classic ACEs measures, parent-child relationships were assessed using the "My hands, my feet and me" questionnaire at age 115 months completed via self-report by the child. The mother's marital status was assessed at the child's age of 10 years and 1 month, with the "You and your surroundings" questionnaire completed by the mother. The number of close friends of the child was measured at age ten using the "Focus at 10" questionnaire, conducted in a focus clinic. Child loneliness at age 9 was assessed using the "Your son/daughter at 9" questionnaire completed by the mother. If the child has been admitted to the hospital has been evaluated at age 57 months, 69 months, 81 months, and 103 months using the "Development and health of my son/daughter", "My school boy/girl", "My son/daughter at school" and "My son/daughter at home and at school" questionnaires, completed by the mother. If there has been a death in the family has been assessed using the "My son/daughter at school" and "My son/daughter at home and at school" questionnaires again. Bullying was measured at age 97 months using the "Me and my school" questionnaire completed by the child. Lastly, school achievement was assessed at age 8 with the "Focus at 8" questionnaire completed by a focus clinic.

We, therefore, matched the AAEs items as follows: Conflict with parents (with conflict with parents), divorce of parents (with mother divorced), health issues (with admission to hospital), educational issues (with educational issues), being bullied (with being bullied), feeling lonely (with loneliness), number of friends (with number of friends) and death to close contact (with death in the family). This selection was based on the availability of items in ALSPAC.

**Results**

We found four clusters in the additional ACEs Network. Cluster 1 included physical and mental abuse and conflict with parents (family relations cluster, Figure 11). Cluster 2 contained items related to social, educational and health issues (social, educational and health factors cluster, Figure 11). Cluster 3 included parental substance abuse items (parental substance abuse cluster, Figure 11). Cluster 4 contained parental factors, such as abuse of mother by partner (parental factors cluster, Figure 11). Therefore, this network contained two more clusters than the original ACEs network. The direct abuse cluster remained, while the family factor clusters were divided into a parental substance abuse and a parental factors cluster. There was a new cluster that contained most of the additional ACE items included in this analysis. Therefore, the clusters remained similar, with a large new cluster containing the additional items (e.g., being bullied or educational issues).

We assessed the relationships between the classic and additional ACEs, mental health, and well-being by estimating the node strength. Emotional abuse inside the family (node 3), physical abuse inside the family (node 1), and emotional abuse outside the family (node 4) showed the highest node strength. These can therefore be considered particularly central to the expanded ACE network. Depression (node 20) and life satisfaction (node 25) showed the highest node strength of the mental health and well-being variables. This result is the same as the original ACEs network. This highlights the robustness of the original findings and underscores the importance of abuse in the family as a central form of abuse during childhood. See Supplementary Materials, Figure 11.

The bridge nodes of the ACEs clusters were death in the family (node 16), followed by emotional abuse inside the family (node 3), admission to hospital (node 15) and mental abuse outside the family (node 4). Death in the family was most strongly related to depression (node 20, r = .11) and drug use (node 23, r = .06). Emotional abuse inside the family was most associated with strongly negatively related to life satisfaction (node 25, r = -.09) and depression (node 20, r = .07). Admission to hospital was most strongly negatively related to depression (node 20, r = -.11) and positively to drug use (node 23, r = .07). Emotional abuse outside the family was most strongly negatively correlated with life satisfaction (node 25, r = -.09) and positively with depression (node 20, r = -.07). The emotional abuse items were included as bridge nodes in the classic ACEs network, but family member's death and hospital admission were additional bridge items in the extended ACEs network. This highlights the particular importance of death in the family and emotional abuse as a gateway to potential mental health issues in later life. See Supplementary Materials, Figure 11.


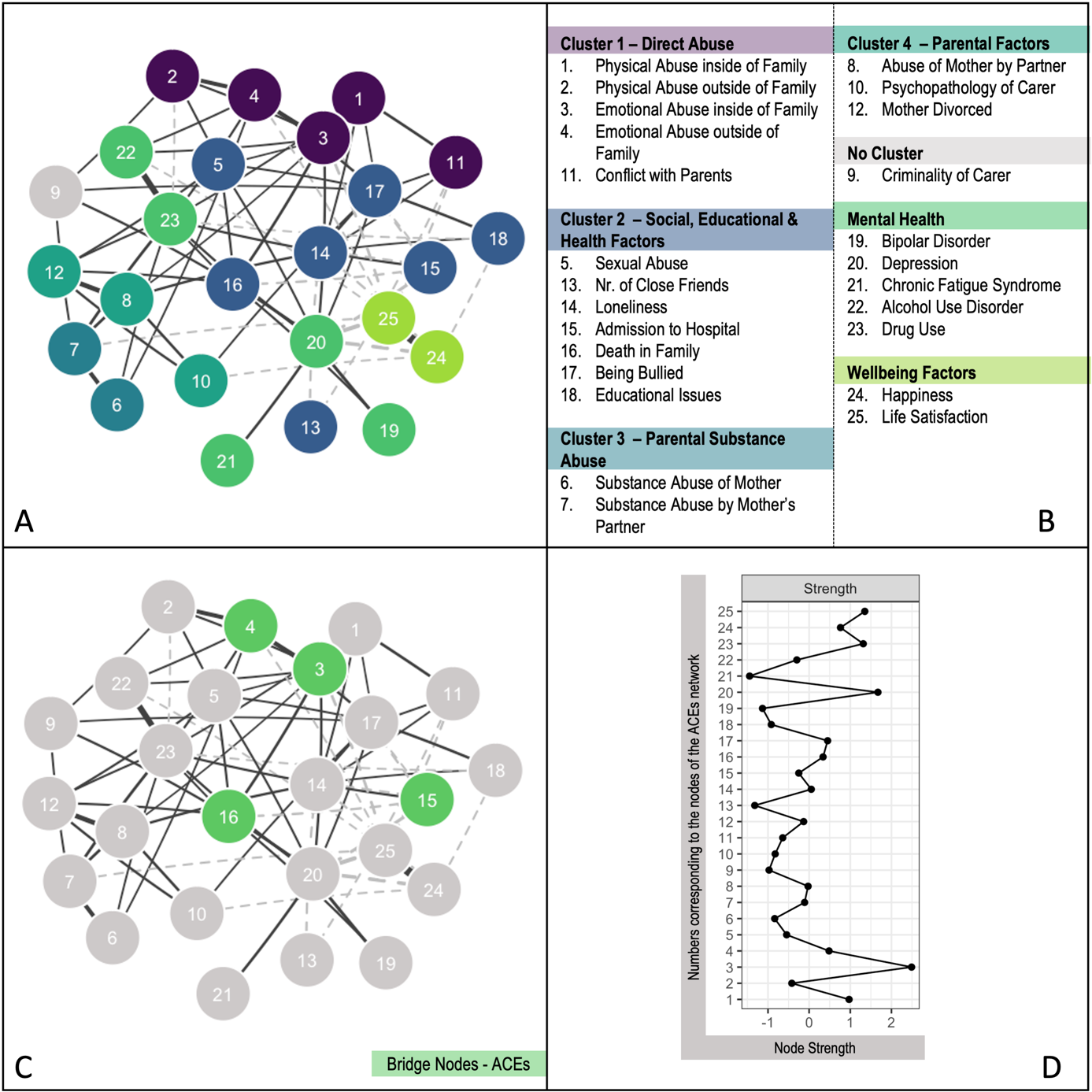


Supplementary Figure 11

A: The expanded ACEs network including four clusters of adversities. Dashed lines represent negative partial correlations, while dark grey lines indicate positive partial correlations. The more saturated the edge, the stronger the partial correlation. B: Network Node Labels. C: Bridge nodes of the adversity cluster (light green). D: Strength scores of the ACEs nodes. Strength refers to the node strength of the ACEs network. Standardized z-scores with numbers corresponding to the ACEs network (see B).
